# Newborn and infant hearing screening outcomes at a tertiary paediatric hospital in the Western Cape, South Africa

**DOI:** 10.4102/sajcd.v73i1.1168

**Published:** 2026-05-19

**Authors:** Silva Kuschke, Cheri van Zyl, Mmakgotso Mokete, Lebogang Ramma

**Affiliations:** 1 Department of Hearing and Speech Sciences, School of Medicine, Vanderbilt University, Nashville, Tennessee, United States; 2 Department of Health and Rehabilitation Sciences, Faculty of Health Sciences, University of Cape Town, Cape Town, South Africa

**Keywords:** newborn and infant hearing screening, otoacoustic emissions, automated auditory brainstem response, risk factors, referral patterns

## Abstract

**Background:**

Despite national guidelines, newborn and infant hearing screening (NIHS) is not universally implemented in South Africa, with scarce data on programme outcomes within the public health sector.

**Objectives:**

To describe NIHS outcomes and implementation patterns at a tertiary paediatric hospital in the Western Cape.

**Method:**

A retrospective record review of the audiology database and hospital records was conducted at Red Cross War Memorial Children’s Hospital between August 2019 and August 2024. Of 7871 children under 6 years, 511 (6.5%) documented NIHS results and were included. Descriptive statistics summarised demographics, risk factors, referral sources, screening methods, and diagnostic outcomes.

**Results:**

Neurological (41.1%) and infectious (25.2%) risk factors related to hearing loss were most prevalent. The mean age of initial screening was 16.3 months, and the mean age of diagnosis was 15.6 months, which is earlier than prior South African reports but substantially later than 1-3-6 benchmark and 2-4-8 benchmark. Most referrals originated from tertiary facilities (60.7%) versus primary healthcare centres (10%). Initial screening (68.7%) employed otoacoustic emissions (45.2% referral rate). Repeat screening, predominantly with automated auditory brainstem response (AABR) (59.9%), reduced referrals to 1.1%. Diagnostic testing (*n* = 110) showed 40% normal hearing, 20% conductive losses, and a predominance of mild to moderate degrees of hearing loss.

**Conclusion:**

Hearing screening in this tertiary setting remains delayed, resource-dependent, and disproportionately tertiary-centred.

**Contribution:**

This study’s results highlight the need to expand primary-level hearing screening, increase AABR use, and adopt standardised protocols to improve timely detection and intervention of hearing loss in South Africa.

## Introduction

Despite published guidelines from the Health Professions Council of South Africa (HPCSA, 2018) on the importance of early hearing detection and intervention (EHDI), standardised implementation of newborn and infant hearing screening (NIHS) in South Africa remains missing to date (Bezuidenhout et al., 2021). The HPCSA guidelines recommend diagnosis of hearing loss and enrolment in early intervention programmes by a maximum of 8 months of age (2018). Although there is growing awareness of the need for EHDI in South Africa, clear legislation mandating NIHS remains absent (Bezuidenhout et al., 2021; De Kock et al., 2016). National surveys conducted in both the private and public healthcare sectors of South Africa suggest screening of only 10% of infants, leaving approximately 90% of newborns with no access to hearing screening (Meyer et al., 2012; Theunissen & Swanepoel, 2008). The public healthcare sector in South Africa services approximately 85% of the general population, but only 7.5% of public facilities offer NIHS services, of which less than 1% offer universal screening (Theunissen & Swanepoel, 2008). Resultantly, reports regarding average age at diagnosis of hearing loss range between 23 months and 44.5 months (Kuschke et al., 2020; Van der Spuy & Pottas, 2008). Late identification of hearing loss implies limited access to early auditory stimulation, which is crucial for age-appropriate speech and language development in children (Olusanya, 2012). Recent evidence continues to demonstrate delayed identification of this abnormality in children. A systematic review by Phanguphangu et al. (2025) reported persistent late detection of congenital hearing loss in South Africa. Studies carried out in KwaZulu-Natal and Gauteng provinces highlight risk-based rather than universal implementation models (Khan & Joseph, 2024; Kanji & Khoza-Shangase, 2021). Community-based pilot programmes suggest that integration into immunisation platforms may be feasible (Kgare & Joubert, 2024; Phanguphangu & Ross, 2025), but large-scale implementation remains limited. Within the Western Cape province, one earlier study profiled childhood hearing loss and associated risk factors (Kuschke et al., 2020), yet limited published data describe contemporary screening outcomes within the public tertiary sector. As South Africa is classified as an upper-middle-income country (World Bank, 2025) with substantial inequities in healthcare access, understanding implementation patterns at tertiary referral centres may provide crucial insights into systemic bottlenecks and opportunities for strengthening early detection pathways. Currently, two NIHS-endorsed techniques exist: Otoacoustic emissions (OAEs) and automated auditory brainstem responses (AABRs) (HPCSA, 2018). The OAE technique measures cochlear outer hair cell functioning and is the most frequently used method for well-baby screening in South Africa (HPCSA, 2018). The AABR method measures neural synchrony of the eighth cranial nerve and lower brainstem and is used for screening in high-risk infants and those who are at risk for auditory neuropathy spectrum disorder (HPCSA, 2018). Evidence is lacking about the status of NIHS programmes in the private and public healthcare sectors in South Africa (Bezuidenhout et al., 2021). Available evidence suggests limited success achieved within existing programmes (Maluleke et al., 2018; Swanepoel et al., 2009). Even in the private healthcare sector of South Africa, where resource constraints are less severe, significant delay is observed in the diagnosis and intervention of childhood hearing loss (Khoza-Shangase et al., 2010; Khoza-Shangase & Kanji, 2021; Meyer et al., 2014). Currently, no legislation mandates the implementation of universal NIHS in South Africa (Bezuidenhout et al., 2021; Khoza-Shangase & Kanji, 2021). Screening is primarily risk-based and targeted at babies and young children who present with known risk factors for hearing loss. Targeted hearing screening is not ideal in contexts where risk factors for hearing loss remain poorly documented (Khoza-Shangase et al., 2017). A recent profile of childhood hearing loss in the Western Cape province revealed that in a sample of 240 children, nearly one-third (27.1%) of those with sensorineural hearing loss (SNHL) had no risk factors, highlighting the need for universal screening coverage (Kuschke et al., 2020). A study conducted in two South African provinces revealed a lack of standardised NIHS implementation at primary, secondary and tertiary levels of public healthcare (Khoza-Shangase et al., 2017). Some of the reasons for the sporadic implementation included limited access to equipment and human resources, financial constraints, and a lack of commitment and political will by the South African government to mandate NIHS. These findings, together with geographical barriers, poverty, long travelling distances and associated costs to attend appointments, have highlighted the need for more context-specific studies regarding NIHS to ensure that contextually relevant strategies are formulated to promote early detection, identification and intervention for hearing loss in children (Khoza-Shangase et al., 2017). This study therefore aimed to describe NIHS outcomes and implementation patterns at a tertiary paediatric hospital in the Western Cape, South Africa.

## Research methods and design

### Study design and population

A retrospective record review of the Audiology Departmental database and patient hospital folders was conducted at Red Cross War Memorial Children’s Hospital (RCWMCH) between August 2019 and August 2024. Purposive sampling based on convenience was employed to select participants under 6 years who had documented NIHS results and, where necessary, diagnostic hearing assessment outcomes covering the study period. In total, 7871 children under 6 years were screened at RCWMCH Audiology. Of these 7871 children, 511 (6.5%) had documented NIHS results and constituted the final study sample.

### Research setting

Red Cross War Memorial Children’s Hospital, located in the Cape Town metropolitan area, is one of only two dedicated paediatric tertiary-level academic hospitals in sub-Saharan Africa. It serves as a central referral hospital for patients across the entire Western Cape province. Therefore, a representative sample of children in the Western Cape is seen at RCWMCH. Children from birth to 13 years of age are served at RCWMCH. The Audiology Department assesses and provides hearing rehabilitation for approximately 250 children per month. Red Cross War Memorial Children’s Hospital does not run its own neonatal hearing screening programme, as infants are not born at RCWMCH.

Referrals are received from various district hospitals, as well as from numerous primary-level clinics and maternal and obstetric units (MOUs). Red Cross War Memorial Children’s Hospital is situated in an upper-middle-income country (World Bank, 2025) and serves mostly children who do not have access to private medical insurance from the public healthcare sector. The typical RCWMCH protocol for initial hearing screening employs OAE equipment if there are no risk factors for retro-cochlear hearing loss. Rescreening is done via the AABR method if a patient is referred through on an initial hearing screening with OAE. If a patient refers for rescreening, diagnostic testing is typically done on the same day, and results for the diagnostic test are documented in the Audiology Departmental database.

### Data collection

This study used an electronic database, which is updated daily in the Department of Audiology at RCWMCH to record patient data, to identify patients who had documented newborn hearing screening results between August 2019 and August 2024. Some data that were not routinely included in the electronic database were captured from clinical records in patients’ hospital files. Documented screening results included those performed at referring facilities prior to tertiary referral, as recorded in clinical notes or referral documentation, as well as those conducted at RCWMCH. In some instances, the age of diagnosis appeared earlier than the recorded age of screening at RCWMCH. The electronic data sheet comprised categorical and numerical data in terms of patient demographic information, NIHS results (initial and re-screen) per ear, type of NIHS equipment that was used, documented risk factors associated with hearing loss, diagnostic assessment results, and geographic area and level of care of referring facilities.

Only four audiologists have access and permission to enter data into the Audiology departmental data sheet, which is stored on a password-protected computer and is backed up every month. To enhance data reliability, all four audiologists responsible for data entry were imparted the necessary training in standardised extraction procedures. Periodic cross-checking of approximately 10% of records was conducted to ensure consistency in data capture. All participant information was captured in a Microsoft Excel spreadsheet, with each participant allocated a unique alphanumeric code with no identifying information used.

### Data analysis

The data collected were imported into a Microsoft Excel 2016 sheet (Microsoft Corp, Redmond, WA, Unites States [US]). Data were analysed using SPSS v.26 (IBM Corp., Armonk, NY, US). Quantitative analysis of data captured on the electronic database was performed using descriptive statistics.

Patient demographics, risk factors associated with hearing loss, geographic distribution and description of referring facilities, method of hearing screening, outcomes of hearing screening, type and degree of hearing loss from diagnostic assessment results were thoroughly analysed and described in terms of percentages of occurrence, mean ages, and standard deviation.

### Ethical considerations

Ethical clearance to conduct this retrospective review study was obtained from the University of Cape Town (HREC441/2022), the Western Cape Department of Health and Wellness (WC 202207_024), and at an institutional level from the RCWMCH Ethics Committee (RCC341). As this was a retrospective study involving routine clinical data, informed consent was not overtly obtained. Caregivers of children accessing care at RCWMCH automatically consent to their anonymised clinical data being used for research purposes when they access services at the tertiary academic hospital-based institution.

## Results

The total number of patients under 6 years who were seen at RCWMCH Audiology from August 2019 to August 2024 was 7871. Of those, only 511 (6.5%) had documented NIHS results, so they were enrolled in the study sample.

### Referral patterns

The majority of children (60.7%) were referred for initial hearing screening by a tertiary-level facility. Only 10% of children were referred by primary healthcare centres. Table 1 shows the referral facilities for the sample.

**TABLE 1:** Referral facilities (*n* = 511).

Referral facility	*n*	%
Tertiary	310	60.7
Secondary	53	10.4
Primary	51	10.0
Maternity and Obstetric Unit	50	9.8
Unknown	27	5.3
Private	20	3.9

### Risk factors associated with hearing loss

Table 2 demonstrates the associated risk factors for hearing loss of the study sample. Neurological (41.1%) and infectious (25.2%) categories accounted for the majority of risk factors for the study sample.

**TABLE 2:** Risk factors associated with hearing loss (*n* = 511).

Risk factor	*n*	%
Neurological†	210	41.1
Infectious‡	129	25.2
Unspecified	83	16.2
Syndromic or genetic§	72	14.1
Not recorded	11	2.2
Ototoxic	4	0.8
Trauma	2	0.4

† , Hypoxic Ischaemic Encephalopathy; Cerebral Palsy; Prematurity;

‡ , Meningitis; Rubella, Cytomegalovirus, Syphilis;

§ , Down Syndrome, Waardenburg Syndrome, Apert Syndrome, Goldenhar Syndrome, Noonan Syndrome.

### Age of suspicion and diagnosis for hearing loss

Age of suspicion and diagnosis were recorded for 92 children in the study sample. There was a 5.5-month mean delay documented in the age of suspicion for hearing loss and the age at diagnosis of hearing loss. Table 3 presents the age at suspicion of hearing loss, the age at diagnosis of hearing loss, and the mean delay from suspicion to diagnosis, in months.

**TABLE 3:** Age of suspicion and diagnosis of hearing loss (*n* = 92).

Age	Mean	s.d.	Range
Suspicion (months)	10.1	16.8	1–70
Diagnosis (months)	15.6	18.4	1–70
Mean delay (months)	5.5	17.7	1–70

s.d., standard deviation.

### Screening outcomes

Initial (*n* = 511) and repeat (*n* = 359) NIHS results were captured in terms of age at initial screening, screening method, and ear-specific results (Table 4). For initial screening, the OAE screening method was used for majority of the sample (68.7%). A remarkably high referral rate (45.2%) was observed on initial NIHS. For repeat screening, the AABR screening method was employed for nearly 60% of the sample, and the referral rate decreased to 1.1%. The high proportion of ‘unknown’ results (17.8%) in the repeat screen group included children for whom diagnostic testing was done immediately after rescreening, and therefore, screening results were not recorded on the Departmental Audiology database.

**TABLE 4:** Initial and repeat newborn and infant hearing screening results (*n* = 511).

Variable	Initial NIHS		Repeat NIHS (*n* = 359)	
	*n*	%	*n*	%
**Screening method**				
OAE	351	68.7	152	42.3
AABR	159	31.1	206	57.4
Unspecified	1	0.2	1	0.3
**Right ear**				
Pass	275	53.8	291	81.1
Refer	220	43.1	4	1.1
Unknown	6	1.2	62	17.8
Could not test	10	1.9	-	-
**Left ear**				
Pass	260	50.9	301	83.8
Refer	231	45.2	4	1.1
Unknown	7	1.4	54	15.1
Could not test	13	2.5	-	-

NIHS, newborn and infant hearing screening; OAE, Otoacoustic emissions; AABR, Automated Auditory Brainstem Response.

### Diagnostic outcomes

Diagnostic hearing results were reviewed for 110 children. Table 5 depicts the diagnostic results in terms of type and degree of hearing loss per ear. Nearly 40% of the sample showed normal hearing bilaterally. Approximately one in five children presented with conductive hearing loss (CHL). Mild and moderate degrees of hearing loss were the most prevalent.

**TABLE 5:** Diagnostic results per ear (*n* = 110).

Hearing loss profile	*n*	%
**Type of hearing loss**		
Right ear:		
Normal	45	40.9
CHL	21	19
SNHL	14	12.7
Mixed hearing loss	1	0.9
Undetermined	23	20.9
ANSD	6	5.5
Left ear:		
Normal	43	39.1
CHL	26	23.6
SNHL	14	12.7
Mixed hearing loss	1	0.9
Undetermined	19	17.3
ANSD	7	6.4
**Degree of hearing loss**		
Right ear:		
Normal	45	40.9
Mild	26	23.6
Moderate	24	21.8
Severe	1	0.9
Profound	8	7.3
ANSD	6	5.5
Left ear:		
Normal	43	39.1
Mild	23	20.9
Moderate	28	25.5
Severe	0	0
Profound	9	8.2
ANSD	7	6.4

CHL, conductive hearing loss; SNHL, sensorineural hearing loss; ANSD, auditory neuropathy spectrum disorder.

## Discussion

The current study provides an updated perspective on the implementation and outcomes of NIHS at a tertiary paediatric hospital in the Western Cape province, South Africa. It evaluates data over a 5-year period. Several key findings highlight both progress and persistent gaps in achieving EHDI benchmarks. The advanced mean age of initial NIHS (16.3 months) emphasises that ‘newborn’ hearing screening in South Africa often occurs well beyond infancy. This delayed occurrence diminishes the opportunity for timely auditory stimulation and speech-language development, thereby highlighting an urgent need for system-level interventions to shift NIHS schedules closer to the neonatal period. The mean age of diagnosis in this cohort, 15.6 months, is earlier than in previous reports from South Africa, in which participants’ age ranged between 18 months and 44.5 months (Kuschke et al., 2020; Swanepoel et al., 2013; Van der Spuy & Pottas, 2008). Nonetheless, this downward trend is encouraging; the current findings remain well outside both the internationally recommended 1-3-6 benchmark (screening by 1 month, diagnosis by 3 months, and intervention by 6 months) and the locally adapted 2-4-8 benchmark proposed for low-income and middle-income contexts (HPCSA, 2018; Olusanya et al., 2014). Analysis of risk factors revealed predominance of neurological (41.1%) and infectious (25.2%) aetiologies in the study sample. These categories included cerebral palsy, hypoxic ischaemic encephalopathy, bacterial meningitis, rubella and cytomegalovirus infections. Such findings mirror the South African epidemiological profile, in which different perinatal complications and infectious diseases remain major contributors to paediatric morbidity (Kehoe, 2025). The significant burden of preventable infections, such as rubella and meningitis, highlights the importance of strengthening vaccination coverage, maternal health programmes, and infection-control practices as complementary strategies to NIHS implementation (Olusanya, 2012). The distribution of referral facilities illustrates another systemic challenge. The majority of children (60.7%) were referred from tertiary facilities, with only 10% referred from primary healthcare centres. This anomalous pattern reflects a disproportionate reliance on higher levels of care, where audiological resources and diagnostic equipment are concentrated, rather than on primary-level MOUs, where universal access could be more effectively achieved. Strengthening NIHS at the primary-care level is essential to reduce inequities in access and ensure earlier identification across the population. Some strategies for strengthening hearing services at the primary care level include training staff on hearing loss risk factors, improved and consistent use of the Road to Health Booklet, early management of middle ear pathology, and clear referral pathways (World Health Organization, 2021). The lifetime economic burden of one annual birth cohort of unidentified infant hearing loss is estimated at R68.6 billion (about R1bn per year of life). The national EHDI programme aligns with national health insurance priorities and supports better educational and employment outcomes (World Health Organization, 2021). Screening outcomes provide further insights into implementation challenges. Nearly half of children (45.2%) were referred on their initial NIHS, yet 40% were later found to have normal bilateral hearing on diagnostic assessment. This discrepancy likely reflects the predominant use of OAE during initial screening, coupled with the older age at which screening occurred. By 16 months of age, middle ear effusions are common, leading to elevated false-positive rates when OAE is employed as the sole screening tool. At repeat screening, where AABR methods were used in approximately 60% of cases, the referral rate decreased considerably to 1.1%. These results corroborate the earlier international literature and validate that AABR is less susceptible to the effects of transient middle ear pathology and environmental noise (Norton et al., 2000; White et al., 2005). The RCWMCH protocol, in which infants who fail OAE screening are rescreened with AABR before their referral for further diagnostics, aligns with best practices and the apparent decreased referral rate on rescreens in this sample. However, the advanced age at initial screening in this cohort diminishes these benefits, because older infants are more vulnerable to otitis media with effusion and other confounding conditions. Diagnostic outcomes also provide important contextual insights. One in five children presented with CHL, thus reflecting the high burden of middle ear disease in low-resource contexts, where recurrent infections and limited access to timely medical care are prevalent (World Health Organization, 2021). Mild and moderate degrees of hearing loss were most common, emphasising the need for greater vigilance in detection, as these degrees can still significantly impact speech and language development if left unmanaged. A notable proportion (20%) of children had an undetermined hearing loss type, which demonstrates the challenges encountered in completing comprehensive diagnostic assessments in young children. Contributing factors likely included fatigue, poor cooperation during behavioural tests, and discharging ears that precluded bone conduction testing. These challenges highlight the importance of strengthening capacity for objective diagnostic measures, such as Auditory brainstem response (ABR), especially in children younger than 3 years or those with complex medical conditions.

The results of this study unravel both progress and persistent gaps in NIHS method in the Western Cape. Though the age of diagnosis is decreasing, the delay in initial screening, the over-representation of tertiary referrals, and the reliance on OAE at older ages all limit the effectiveness of current approaches. These results align with some previous South African studies that describe sporadic, resource-dependent, and non-standardised implementation of NBHS (Khoza-Shangase et al., 2017; Maluleke et al., 2018). Therefore, achieving meaningful improvement will require a shift towards universal screening at the primary level, increased use of AABR in high-risk or older infants, and parallel strengthening of public health measures that address infectious and perinatal risk factors.

### Policy implications and future research

National policy reform is needed to standardise NIHS protocols, strengthen accountability mechanisms, and support equitable equipment distribution in South Africa. Integration of hearing screening into existing maternal and child health platforms, including immunisation visits, should be formally considered. Future research should prioritise implementation trials at the primary care level and cost-effectiveness analyses to inform scalable national policy decisions and achieve the desired outcomes.

## Conclusion

Although the mean age of diagnosis was lower in the current study than previously reported in South Africa, it remains substantially delayed compared to international and local EHDI benchmarks. A high proportion of referrals originated from tertiary facilities, revealing inherent weaknesses in the integration of NIHS at the primary care level, where universal access is most feasible. Infectious and neurological risk factors are the most common contributors to hearing loss, consistent with South Africa’s broader child health context. Screening outcomes revealed a high initial referral rate when the OAE approach was used at older ages, but this was mitigated by subsequent AABR rescreening. These findings highlight both encouraging trends and ongoing barriers. To move closer to timely identification and intervention, South Africa requires standardised NIHS protocols, stronger integration of screening at the primary care level, greater use of AABR technology, and complementary strategies to reduce preventable infectious and perinatal risk factors. Overall, we can conclude that sustainable system-level investment and policy support are needed to ensure equitable access to EHDI for all children.

## References

[CIT0001] Bezuidenhout, J.K., Khoza-Shangase, K., De Maayer, T., & Strehlau, R. (2021). Outcomes of newborn hearing screening at an academic secondary level hospital in Johannesburg, South Africa. *South African Journal of Communication Disorders*, 68(1), e1–e8. 10.4102/sajcd.v68i1.741 PMC7876983 33567828

[CIT0002] De Kock, T., Swanepoel, D., & Hall III, J.W. (2016). Newborn hearing screening at a community-based obstetric unit: Screening and diagnostic outcomes. *International Journal of Pediatric Otorhinolaryngology*, 84, 124–131. 10.1016/j.ijporl.2016.02.031 27063767

[CIT0003] Health Professions Council of South Africa (HPCSA). (2018). *Professional board for speech, language and hearing professions: Early hearing detection and intervention (EHDI) guidelines*. HPCSA.

[CIT0004] Kanji, A., & Khoza-Shangase, K. (2021). Risk factors for hearing impairment in neonates in South Africa: Scoping the context for newborn hearing screening planning. *The Journal of Maternal-Fetal & Neonatal Medicine*, 34(13), 2107–2116. 10.1080/14767058.2019.1658732 31434520

[CIT0005] Kehoe, K. (2025). Burden and causes of ongoing paediatric infectious disease morbidity and mortality in the Western Cape Province of South Africa [PhD thesis]. Retrieved from open.uct.ac.za

[CIT0006] Kgare, K.S., & Joubert, K. (2024). Community-based infant hearing screening: Outcomes of a rural pilot programme. *South African Journal of Communication Disorders*, 71(1), 1045. 10.4102/sajcd.v71i1.1045 PMC11538158 39494639

[CIT0007] Khan, N.B., & Joseph, L. (2024). Risk factors and hearing outcomes in infants and young children in KwaZulu-Natal, South Africa. *South African Journal of Communication Disorders*, 71(1), 1–11. 10.4102/sajcd.v71i1.1031 PMC11369661 39221742

[CIT0008] Khoza-Shangase, K., Barratt, J., & Jonosky, J. (2010). Protocols for early audiology intervention services: Views from early intervention practitioners in a developing country. *South African Journal of Child Health*, 4(4), 100–105. Retrieved from https://www.ajol.info/index.php/sajchh/article/view/69993

[CIT0009] Khoza-Shangase, K., & Kanji, A. (2021). Best and next practice in South Africa for early hearing detection and intervention. In K. Khoza-Shangase & A. Kanji (Eds.), *Early detection and intervention in Audiology: An African perspective* (pp. 264–278). Wits University Press.

[CIT0010] Khoza-Shangase, K., Kanji, A., Petrocchi-Bartal, L., & Farr, K. (2017). Infant hearing screening from a developing country context: Status from two South African provinces. *South African Journal of Child Health*, 11(4),159–163. Retrieved from https://hdl.handle.net/10520/EJC-cb936e79a

[CIT0011] Kuschke, S., Swanepoel, D.W., Le Roux, T., & Strauss, S. (2020). Profile of childhood hearing loss in the Western Cape, South Africa. *International Journal of Pediatric Otorhinolaryngology*, 137, 110248. 10.1016/j.ijporl.2020.110248 32658802

[CIT0012] Maluleke, N.P., Khoza-Shangase, K., & Kanji, A. (2018). Hearing impairment detection and intervention in children from centre-based early intervention programmes. *Journal of Child Health Care*, 23(2), 1–10. 10.1177/1367493518788477 30068223

[CIT0013] Meyer, M., Swanepoel, D.W., & Le Roux, T. (2014). National survey of pediatric audiological services for diagnosis and intervention in the South African private health care sector. *South African Journal of Communication Disorders*, 61(1), E1–E8. 10.4102/sajcd.v61i1.62 26305440

[CIT0014] Meyer, M.E., Swanepoel, D.W., Le Roux, T., & Van der Linde, M. (2012). Early detection of infant hearing loss in the private health care sector of South Africa. *International Journal of Pediatric Otorhinolaryngology*, 76(5), 698–703. 10.1016/j.ijporl.2012.02.023 22386272

[CIT0015] Norton, S.J., Gorga, M.P., Widen, J.E., Folsom, R.C., Sininger, Y., Cone-Wesson, B., Vohr, B.R., & Fletcher, K.A. (2000). Identification of neonatal hearing impairment: Summary and recommendations. *Ear and Hearing*, 21(5), 529–535. 10.1097/00003446-200010000-00014 11059708

[CIT0016] Olusanya, B.O. (2012). Neonatal hearing screening and intervention in resource-limited settings: An overview. *Archives of Disease in Childhood*, 97, 654–659. 10.1136/archdischild-2012-301786 22611062

[CIT0017] Olusanya, B.O., Neumann, K.J., & Saunders, J.E. (2014). The global burden of disabling hearing impairment: A call to action. *Bulletin of the World Health Organization*, 92, 367–373. 10.2471/blt.13.128728 24839326 PMC4007124

[CIT0018] Phanguphangu, M., Kgare, K., & Ross, A.J. (2025). Age of detection of congenital hearing loss in South Africa: A systematic review. *Journal of Public Health in Africa*, 16(1), 777. 10.4102/jphia.v16i1.777 40475230 PMC12138633

[CIT0019] Phanguphangu, M., & Ross, A.J. (2025). Feasibility of integrating nurse-administered infant hearing screening into an immunisation programme at a primary healthcare clinic in South Africa: A Hybrid Type 2 trial. *International Journal of Pediatric Otorhinolaryngology*, 195, 112446. 10.1016/j.ijporl.2025.112446 40578245

[CIT0020] Swanepoel, D., Storbeck, C., & Friedland, P. (2009). Early hearing detection and intervention in South Africa. *International Journal of Pediatric Otorhinolaryngology*, 73(6), 783–786. 10.1016/j.ijporl.2009.01.007 19187975

[CIT0021] Swanepoel, D.W., Johl, L., & Pienaar, D. (2013). Childhood hearing loss and risk profile in a South African population. *International Journal of Pediatric Otorhinolaryngology*, 77(3), 394–398. 10.1016/j.ijporl.2012.11.034 23266158

[CIT0022] Theunissen, M., & Swanepoel, D.W. (2008). Early hearing detection and intervention services in the public health sector in South Africa. *International Journal of Audiology*, 47(1), S23–S29. 10.1080/14992020802294032 18781510

[CIT0023] Van der Spuy, T., & Pottas, L. (2008). Infant hearing loss in South Africa: Age of intervention and parental needs for support. *International Journal of Audiology*, 47(sup1), S30–S35. 10.1080/14992020802286210 18781511

[CIT0024] White, K.R., Vohr, B.R., Meyer, S., Widen, J.E., Johnson, J.L., Gravel, J.S., James, M., Kennalley, T., Maxon, A.B., Spivak, L., Sullivan-Mahoney, M., & Weirather, Y. (2005). A multisite study to examine the efficacy of the otoacoustic emission/automated auditory brainstem response newborn hearing screening protocol. *American Journal of Audiology*, 14(2), S178–S185. 10.1044/1059-0889(2005/020) 16489862

[CIT0025] World Bank. (2025). *Global Economic Prospects, June 2025*. World Bank.

[CIT0026] World Health Organization. (2021). *World report on hearing*. World Health Organization.

